# Kidney–heart crosstalk: the extracellular vesicles connection

**DOI:** 10.1093/ndt/gfag022

**Published:** 2026-02-09

**Authors:** Carmine Zoccali, José Manuel Valdivielso, Liffert Vogt, Evangelia Ntounousi, Fotini Iatridi, Nejc Piko, Beatriz Fernández-Fernández, Alberto Ortiz, Shanmugakumar Chinnappa, Patrick B Mark, Mehmet Kanbay, Francesca Mallamaci, Andrzej Wiecek, Benedetta Bussolati, Roel Bijkerk

**Affiliations:** Renal Research Institute, New York, USA; Institute of Molecular Biology and Genetics (Biogem), Ariano Irpino, Italy; Associazione Ipertensione Nefrologia Trapianto (IPNET), c/o Nefrologia, Grande Ospedale Metropolitano, Reggio Calabria, Italy; Institut de Recerca Biomedica de Lleida – Fundacio Dr Pifarre, IRBLleida, Lleida, Spain; RICORS 2040, Instituto de Salud Carlos III, Madrid, Spain; Department of Internal Medicine, section Nephrology, Amsterdam University Medical Center, University of Amsterdam, Amsterdam, The Netherlands; Department of Nephrology, University Hospital of Ioannina, Ioannina, Greece; First Department of Nephrology, Hippokration Hospital, Aristotle University of Thessaloniki, Thessaloniki, Greece; Department of Dialysis, Clinic for Internal Medicine, University Medical Centre Maribor, Maribor, Slovenia; 2IIS-Fundacion Jimenez Diaz UAM, Madrid, Spain; Department of Medicine, School of Medicine, Universidad Autónoma de Madrid, Madrid, Spain; 2IIS-Fundacion Jimenez Diaz UAM, Madrid, Spain; Department of Nephrology, Doncaster and Bassetlaw Teaching Hospitals NHS Trust, Doncaster, UK; Leeds Institute of Cardiovascular and Metabolic Medicine (LICAMM), University of Leeds, Leeds, UK; School of Cardiovascular and Metabolic Health, University of Glasgow, Glasgow, UK; Division of Nephrology, Department of Internal Medicine, Koc University, School of Medicine, Istanbul, Turkey; Associazione Ipertensione Nefrologia Trapianto (IPNET), c/o Nefrologia, Grande Ospedale Metropolitano, Reggio Calabria, Italy; Department of Nephrology, Transplantation and Internal Medicine, Medical University of Silesia, Katowice, Poland; Department of Molecular Biotechnology and Health Science, University of Turin, Turin, Italy; Division of Nephrology and the Einthoven Laboratory for Vascular and Regenerative Medicine, Department of Internal Medicine, Leiden University Medical Center, Leiden, The Netherlands

**Keywords:** cardiovascular disease, chronic kidney disease, extracellular vesicles

## Abstract

Chronic kidney disease (CKD) is a major public health concern, closely linked to an increased risk of cardiovascular disease (CVD), which remains the leading cause of morbidity and mortality in this population. While traditional risk factors such as hypertension and diabetes are prevalent in CKD, disease-specific mechanisms—including chronic inflammation, oxidative stress, mineral disturbances and the accumulation of uraemic toxins—further amplify cardiovascular vulnerability. In CKD, both the abundance and molecular cargo of circulating extracellular vesicles (EVs) are altered, reflecting the underlying metabolic and inflammatory milieu. These EVs propagate endothelial dysfunction, vascular calcification, inflammation, thrombosis and cardiac remodelling by transferring bioactive molecules such as proteins and microRNAs to target cells. Emerging evidence suggests that EVs not only serve as biomarkers for early detection and risk stratification of CVD in CKD but may also represent novel therapeutic targets. Preclinical studies demonstrate the potential of stem cell–derived and engineered EVs to promote cardiac repair and modulate pathological signalling. However, translation into clinical practice requires rigorous standardization, safety validation and well-designed human trials. This review synthesizes current knowledge on the mechanisms by which EVs bridge renal dysfunction and cardiovascular pathology, discusses their utility as biomarkers and outlines a research agenda for harnessing their therapeutic potential in CKD-associated CVD.

## INTRODUCTION

On a world scale, chronic kidney disease (CKD) affects an estimated 10%–15% of adults, and its presence dramatically increases the likelihood of cardiovascular events [1]. Notably, the risk of cardiovascular disease (CVD) rises in parallel with declining kidney function, even in the earliest stages of CKD. For many patients, the threat of CVD far outweighs the risk of progressing to end-stage renal disease, making CVD the leading cause of morbidity and mortality among those with impaired renal function. This heightened risk is evident even with mild reductions in estimated glomerular filtration rate (eGFR) or the presence of albuminuria, underscoring the need for vigilance even in early CKD [2].

The drivers of cardiovascular risk in CKD are multifaceted, reflecting a convergence of traditional and CKD-specific factors [2]. CKD introduces a spectrum of non-traditional, disease-specific risk factors that further amplify cardiovascular vulnerability. Chronic low-grade inflammation and increased oxidative stress are hallmarks of CKD, promoting endothelial dysfunction and accelerating atherogenesis [3, 4]. Disturbances in mineral and bone metabolism—manifesting as abnormalities in calcium, phosphate and parathyroid hormone—lead to vascular calcification and increased arterial stiffness [5]. The accumulation of uraemic toxins [6], mainly a direct consequence of impaired renal clearance, exerts toxic effects on the cardiovascular system. At the same time, anaemia, frequently observed in CKD, increases cardiac workload and fosters structural heart changes such as left ventricular hypertrophy [7]. Additional contributors, including volume overload from sodium and water retention and heightened sympathetic nervous system activity, further predispose individuals to arrhythmias and heart failure [4]. Despite notable advances in our understanding and management of cardiovascular risk in CKD, cardiovascular mortality remains unacceptably high in the CKD population [8]. For this reason, there is a need for new research that delves deeper into the unique pathophysiology of CVD in CKD.

Extracellular vesicles (EVs) represent an important area for advancing pathophysiological knowledge in CKD, because they are key mediators of cross-talk between the kidney and other organs, particularly the cardiovascular system [9]. Various alterations in EVs have been found in CKD patients [10, 11], and these alterations are intensively investigated as potential mediators of renal damage [10] and its complications [12]. However, the effect of EVs on CVD in CKD has been scarcely studied.

Herein, we briefly recapitulate the biology of EVs and their role in the control of cardiovascular function. We then focus on EV-dependent mechanisms with potential value for understanding the pathophysiology of CVD in CKD, to eventually outline a research roadmap to explore the therapeutic potential of EVs for the prevention and treatment of CVD in CKD.

## BIOLOGY OF EVs: BIOGENESIS, CLASSIFICATION AND FUNCTION

**Figure 1:**
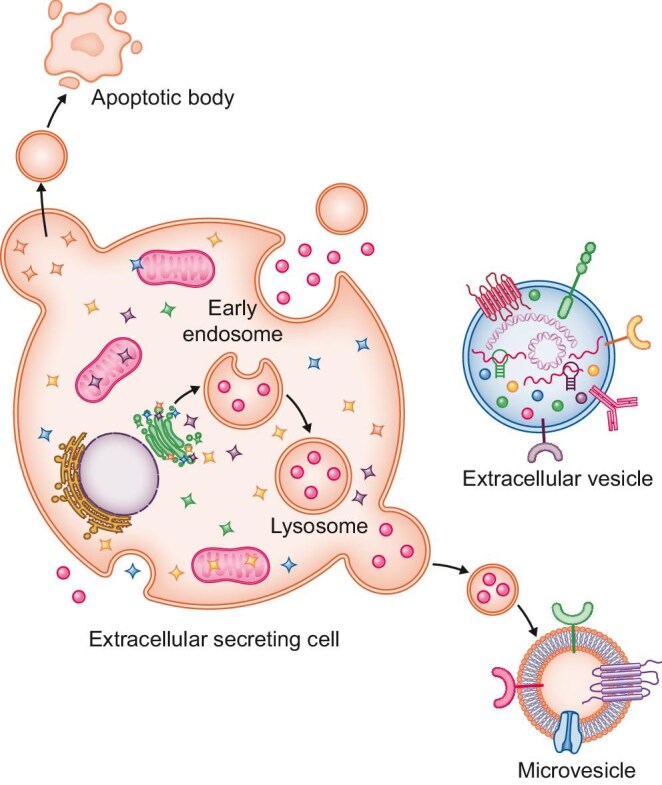
EVs are cell-derived particles filled with bioactive molecules—proteins, nucleic acids and lipids—that form their structure while carrying distinctive cellular signatures. Cells throughout the human body use EVs as a communication system, releasing them into body fluids to exchange information and materials with neighbouring and distant tissues. Stem cells are particularly prolific EV producers in humans. The figure depicts an EV, the generation of a microvesicle, an exosome and an apoptotic body.

EVs are a heterogeneous group of membrane-bound nanoparticles released by virtually all cell types [13] (Fig. 1). They have emerged as critical mediators of intercellular communication, capable of transferring a diverse array of bioactive molecules—including proteins, lipids, messenger and non-coding RNAs, DNA fragments and metabolites—to recipient cells, thereby modulating cellular phenotype and function. The field of EV research is an expanding phase. However, the rich literature has been complicated by inconsistent terminology and a lack of standardized biomarkers [14]. To address this issue, the International Society for Extracellular Vesicles (ISEV) has recommended using ‘extracellular vesicle’ as a generic term and has established minimal experimental standards to improve reproducibility and clarity in the field [14].

Based on their biogenesis pathways, EVs have been classified into three major subtypes: exosomes, microvesicles and apoptotic bodies. Exosomes are formed through the endosomal pathway, specifically by the inward budding of the endosomal membrane to create multivesicular bodies (MVBs), which then fuse with the plasma membrane to release their contents. Microvesicles, in contrast, are produced by direct outward budding from the plasma membrane, while apoptotic bodies are released during programmed cell death (Fig. 1). Apoptosis is a physiological form of cell death that suppresses inflammation, and apoptotic bodies display ‘eat-me’ signals on their surface that trigger rapid engulfment by adjacent cells. Other forms of programmed cell death (ferroptosis, necroptosis, pyroptosis) are proinflammatory and immunogenic [15].

A combination of various factors facilitates the release of EVs [16]. When cells are activated by stimuli such as cytokines, growth factors or immune signals, or when they experience stress (such as hypoxia or oxidative stress), EV production and release increase. A rise in intracellular calcium levels acts as a key trigger, activating enzymes that disrupt membrane stability and promote vesicle budding. The reorganization of the actin cytoskeleton is also crucial, as it enables the physical processes required for vesicle formation and release. Changes in the lipid composition of the cell membrane, particularly the accumulation of ceramide and the externalization of phosphatidylserine, facilitate membrane curvature and vesicle budding. Specific proteins, including Ras-like protein from rat brain (Rab), and the GTP-binding proteins (Rab GTPases) and Soluble N-ethylmaleimide-sensitive factor Attachment protein REceptors (SNAREs), play essential roles in the trafficking and fusion of vesicle-containing compartments with the plasma membrane, allowing for the release of EVs. The local pH and ionic environment, such as acidic conditions or shifts in potassium levels, can further modulate this process. The inherent properties of the cell, including its type and physiological or pathological state, influence both the quantity and the characteristics of EVs released. Additionally, external factors, such as exposure to drugs, toxins, pathogens or mechanical forces, such as shear stress [17], can enhance EV release by altering cellular machinery or inducing stress responses. All these factors interact to regulate the complex and dynamic process of extracellular vesicle release.

## EVs IN CARDIOVASCULAR PATHOPHYSIOLOGY

EVs are released by virtually all cells, including those of the cardiovascular system, such as cardiomyocytes, endothelial cells, fibroblasts, platelets, vascular smooth muscle cells (VSMCs), leucocytes, monocytes and macrophages [18]. These cell-released vesicles are relevant for maintaining normal cardiac structure and function under physiological conditions. A large body of evidence suggests that the effects of EVs on target cells are mainly dependent on cargoes such as proteins, enzymes and non-coding RNAs [19] [miRNAs, long non-coding RNAs and circular RNAs (circRNAs)]. Depending on the condition of the source cells, EVs carrying specific miRNAs and proteins can regulate diverse functions in target cells, maintaining cardiovascular homeostasis or, conversely, contributing to disease progression in CVDs [18].

EVs and their miRs are key players in mediating inflammatory and coagulative responses, thereby contributing to angiogenesis and the development of atherosclerosis [20]. Under disease conditions such as atherosclerosis, diabetes or myocardial infarction (MI), EVs are, in fact, enriched in pro-inflammatory and pro-coagulant molecules, amplifying vascular injury [21]. For example, EVs derived from activated endothelial cells and monocytes, particularly those expressing cell adhesion and procoagulant molecules, associate with early vascular dysfunction [22]. Moreover, cardiac fibroblast-derived EVs enriched in miR-21-3p act as paracrine-signalling mediators of cardiomyocyte hypertrophy [23] supporting the EV-mediated cross-talk between cardiomyocytes and fibroblasts in cardiac remodelling.

Adverse cardiovascular effects can be induced, for instance, via macrophage-derived foam cells. Indeed, these cells can promote VSMC migration and adhesion, a process mediated by the integration of EVs into the VSMCs and the subsequent activation of extracellular-regulated protein kinase (ERK) and protein kinase B (PKB) [24]. M1-like macrophage-derived EVs were also reported to suppress angiogenesis and exacerbate cardiac dysfunction in an MI microenvironment [25].

Activated platelets contribute further to this complexity by releasing abundant EVs containing metabolites and non-coding RNAs, such as circRNAs [26], implicated in the VSMC phenotype linked to atherosclerosis development [27]. Interestingly, in pathophysiological conditions characterized by platelet activation, e.g. type 2 diabetes, aspirin may reduce circulating miR-126 [28] levels. Adding to previous examples, endothelial and platelet-derived EVs, increase in patients with stable coronary artery disease and atherosclerotic conditions, and contain microRNA-92a-3p, which can regulate angiogenesis and contribute to vascular dysfunction [21]. Adipocyte-derived EVs promote plaque burden and exacerbate vulnerable atherosclerosis by inducing angiogenesis of the vasa vasorum in diabetic ApoE^–/–^ mice [29]. Visceral fat-derived EVs, regardless of obesity, also facilitate macrophage-derived foam cell formation by inhibiting cholesterol efflux through ABCA1 and ABCG1 transporters [30]. In addition, EVs released from hypertrophic adipocytes can impair endothelial function and promote the development of atherosclerosis, while those from diabetic cardiomyocytes inhibit endothelial cell proliferation and angiogenesis [31].

EVs subtypes—particularly CD14⁺ monocyte‑derived, CD41⁺/CD61⁺ platelet‑derived and CD146⁺ endothelial vesicles, as well as cardiac miRNA‑enriched exosome‑like vesicles—show biologically plausible associations with incident cardiovascular events and treatment response, for example in the ASCOT‑LLA nested case–control study of hypertensive patients, where higher CD14⁺ EVs predicted major adverse cardiovascular events and CD146⁺ EVs fell with atorvastatin therapy [32]. Across coronary artery disease, atrial fibrillation and heart failure, multiple small studies and recent reviews report altered numbers and cargo of circulating EVs and highlight their potential as diagnostic and prognostic markers [33]. However, rigorous evaluation of added value over guideline‑based scores—formal calibration, robust c‑statistics, and clinically meaningful risk reclassification with internal and external validation—is largely lacking, so EVs remain experimental prognostic biomarkers.

EVs are not solely agents of harm. Their protective roles are equally well documented, particularly in the context of tissue repair and regeneration. *In vitro* studies [34] support a protective role of EVs in the interaction of endothelial cells and pro-inflammatory cells. Endothelial cells exposed to inflammatory stimuli upregulate intercellular adhesion molecule 1 (ICAM-1) expression and promote endothelial–monocyte adhesion. However, endothelial EVs can exert counterbalancing and anti-inflammatory effects by transferring miR-222 to recipient cells, thereby reducing ICAM-1 expression [20]. Similarly, endothelial EVs-mediated transfer of miR-126 promotes the repair of endothelial damage caused by hyperglycaemic conditions, and, *in vivo*, the administration of miR-126-enriched endothelial EVs accelerates reendothelialization after endothelial injury [35].

Administration of endothelial EVs containing miR-126, has been shown to promote vascular repair and reendothelialization after injury, and to accelerate endothelial healing *in vivo* [36]. In humans, coronary serum EVs isolated from patients with myocardial ischaemia promote angiogenesis via a nitric oxide signalling pathway [37], a potentially protective effect.

In a therapeutic perspective, mesenchymal stromal cell (MSC)-derived EVs are especially notable for their cardioprotective effects: they can attenuate myocardial ischaemia–reperfusion injury, stimulate angiogenesis, reduce apoptosis and improve cardiac function after infarction. In animal models, engineered EVs or those loaded with specific microRNAs have been used to restore cardiac function and reduce adverse remodelling after MI. MSC-derived (EVs-carried) microRNAs were also reported to display protective effects in myocardial ischaemia–reperfusion injury in mice by shuttling miR-182, which modifies the polarization status of macrophages [38]. Moreover, hypoxia-induced MSC-derived EVs facilitate ischaemic cardiac repair through miR-125b-mediated amelioration of cardiomyocyte apoptosis and prevention of cardiomyocyte death in MI [39]. In parallel, MiR-93-5p-enhanced adipose MSC-EVs prevent cardiac injury by inhibiting autophagy and inflammation, and attenuate acute MI-induced myocardial damage [40]. Donor-derived EVs have a crucial role in triggering alloimmune responses, ultimately contributing to transplant rejection [41]. On the other hand, in liver transplantation EVs enriched in PD‑L1 under immunosuppressive regimens can promote T‑cell anergy and graft tolerance [42].

In summary, EVs have emerged as pivotal mediators in cardiovascular biology, exerting both detrimental and protective effects depending on their cellular origin, molecular cargo, and the physiological or pathological context. In particular, cardiovascular risk factors alter the composition and signalling potential of circulating EVs, influencing myocardial injury [19] and post-infarction remodelling, making EVs both mediators and modulators of cardiovascular pathology and repair [43]. This also suggests a significant potential for interventions by increasing or decreasing miR-containing EVs [43].

Thus, the impact of EVs on cardiovascular integrity is fundamentally context-dependent. EVs can exert favourable, neutral or unfavourable effects on target cells, including modulating gene expression, influencing cell phenotype and molecular pathways, and mediating biological functions. This dual nature means that while EVs can propagate vascular injury and inflammation under disease conditions, they also hold tremendous potential as vehicles for cardiovascular repair and as therapeutic agents.

## EXTRACELLULAR VESICLES AS POTENTIAL MEDIATORS OF CVD IN CKD (BOX 1)

On 15 August 2025, we made a PubMed search using the terms ‘(‘Extracellular vesicles’ OR exosomes OR microvesicles) AND (‘Cardiovascular disease’ OR ‘cardiovascular risk’ OR CVD) AND (‘Chronic kidney disease’ OR CKD) AND (Humans[Mesh]) NOT (Review[Publication Type])’. This search yielded 21 original *in vitro* or clinical papers, which we briefly summarize in Table 1 and discuss further below. Notably, no robust intervention study has yet tested the potential impact of EVs on clinical outcomes in CKD. A placebo-controlled, randomized study tested the effect of two doses of cell-free cord-blood MSC, administered intravenously 1 week apart, in stage 3–4 CKD patients (20 treated with MSC and 20 receiving a placebo) [44]. The intervention was safe, and MSC-treated patients had a marked but transient improvement of eGFR from a baseline value of 31 ± 4 mL/min/1.73 m^2^ to 47 ± 5 mL/min/m^2^ at 12 weeks (*P* ≤ .009) and to 41 ± 3 mL/min/1.73 m^2^ at 1 year, which was not statistically significant. These changes were paralleled by an improvement in albuminuria, which did not reach statistical significance. At 1 year, patients of the treatment group also exhibited an increase in plasma transforming growth factor (TGF)-β1 (*P* ≤ .0035), which was mirrored by a decrease in plasma tumour necrosis factor (TNF)-α (*P* < .009).

Box 1. **Extracellular vesicles in CKD-associated cardiovascular pathophysiology.**

**EV-mediated endothelial dysfunction, inflammation and atherosclerosis**

EVs are released by virtually all cardiovascular and immune cells, including endothelial cells, platelets, leukocytes, monocytes, macrophages, cardiomyocytes, fibroblasts and VSMCs, and are essential for intercellular communication in health and disease [18–22]. Their cargo—particularly non-coding RNAs (miRNAs, lncRNAs, circRNAs) and proteins—changes with the activation state of the parent cell and can either preserve vascular homeostasis or promote inflammation, thrombosis and atherosclerosis [19, 20, 22–24]. In CKD (including diabetic nephropathy and paediatric CKD), uraemic toxins, oxidative stress and chronic low-grade inflammation drive the release of endothelial- and platelet-derived EVs enriched in adhesion molecules, pro-coagulant factors and pro-inflammatory miRNAs, which amplify endothelial activation, monocyte adhesion and early vascular dysfunction [21, 22, 45–52, 50, 60, 61].

**EVs in vascular calcification and arterial stiffness**

Disturbances in mineral metabolism and the CKD inflammatory milieu promote the release of calcifying EVs from VSMCs. These EVs are enriched in annexins, alkaline phosphatase, fetuin-A and specific miRNAs, and act as nucleation sites for hydroxyapatite deposition within the arterial media, increasing vascular stiffness and left ventricular afterload [57, 58]. Experimental CKD models show that activation of IKK2/NF-κB in VSMCs or inhibition of epidermal growth factor receptor signalling reduces the production of apoptotic, calcifying EVs and attenuates vascular calcification, highlighting calcifying EVs as central effectors and therapeutic targets in CKD vasculopathy [55, 57, 58]. Clinical studies in advanced CKD and dialysis patients support a similar pro-calcific EV profile, with monocyte- and platelet-derived EVs and altered EV-miRNA signatures associating with vascular damage and arterial stiffness [50–52, 59, 74, 75].

**EVs in cardiac remodelling and uraemic cardiomyopathy**

Cardiac fibroblast- and cardiomyocyte-derived EVs shuttle miRNAs and proteins that modulate hypertrophy, fibrosis and angiogenesis in the myocardium. Under atherosclerotic or CKD-like conditions, fibroblast-derived exosomes enriched in miR-21-3p and macrophage- or adipocyte-derived EVs can drive cardiomyocyte hypertrophy, microvascular rarefaction and adverse remodelling [23–27, 29–31]. Experimental data indicate that EVs from CKD patients alter gene expression and mitochondrial function in cardiac cells, promoting the phenotype of uraemic cardiomyopathy [62]. Conversely, coronary serum EVs from patients with myocardial ischaemia and certain endothelial EVs can stimulate angiogenesis via nitric oxide–dependent signalling, suggesting context-dependent adaptive responses [37].

**Protective and reparative EVs and therapeutic potential**

Not all EVs are detrimental: endothelial-derived EVs carrying miR-222 or miR-126 can dampen inflammatory activation and promote endothelial repair and re-endothelialization after injury [34–36]. In preclinical models of MI and ischaemia–reperfusion injury, MSC-derived EVs and engineered EVs enriched in specific miRNAs (e.g. miR-182, miR-125b, miR-93-5p) attenuate cardiomyocyte apoptosis, improve post-infarction remodelling and enhance cardiac function [38–40]. In CKD and ESKD, dialysis modality and established cardiovascular drugs such as statins, aspirin and sodium-glucose cotransporter 2 inhibitors modulate EV biogenesis and cargo, suggesting that part of their cardiovascular benefit (or harm) may be mediated through EV pathways [28, 30, 41, 59, 68–70, 77]. Together, these findings support EVs both as mediators of CKD-associated cardiovascular damage (endothelial dysfunction, calcification, inflammation, thrombosis, remodelling) and as promising vehicles or targets for future therapeutic interventions.

**Table 1:** Preclinical studies are listed first, followed by clinical studies, to distinguish mechanistic from patient-based evidence.

First author [Ref.]	Study type	Population/model	EVs/miRNAs assessed	Cardiovascular/vascular relevance in CKD
Figuer A *et al*. [46]	Preclinical, *in vitro*	Human endothelial cells exposed to indoxyl sulfate	EC-derived EVs; proteomic profile	Indoxyl sulfate induces pro-inflammatory and adipogenesis-related proteins in ECs and in their EVs, supporting a role for uraemic toxin–driven EC-EVs in endothelial dysfunction characteristic of CKD
Zhang Y *et al*. [53]	Preclinical, rats with streptozocin-induced diabetes	Effect of platelet microparticles on glomerular endothelial injury in early diabetic nephropathy and primary rat glomerular endothelial cells	Platelet microparticles; EC	Findings in this study demonstrate a pathogenic role of platelet microparticles in glomerular endothelium dysfunction, and suggest a potential therapeutic target, CXCL7, for treatment of early diabetic nephropathy
Miyazaki-Anzai S *et al*. [58]	Preclinical, *in vivo* CKD model	CKD animals with vascular calcification	VSMC-derived calcifying EVs	Activation of IKK2/NF-κB in VSMCs reduces apoptotic, calcifying EVs and vascular stiffness, implicating smooth-muscle EVs as effectors and potential targets in CKD-induced vascular calcification
Bakhshian Nik A *et al*. [59]	Preclinical, *in vivo* and *in vitro* CKD models	CKD-associated vascular calcification	Vascular calcifying EVs; EGFR-dependent biogenesis	EGFR inhibition decreases biogenesis of calcifying EVs and attenuates vascular calcification, identifying EGFR-regulated EVs as therapeutic targets in CKD vasculopathy
Lupo MG *et al*. [75]	Preclinical, *in vivo* CKD model	CKD animals with arterial medial calcification	VSMC-derived, calcium- and ALP-rich EVs (PCSK9-dependent)	PCSK9 promotes a pro-calcific smooth-muscle phenotype and EV release, driving medial calcification and linking lipid pathways to EV-mediated vascular damage in CKD
Nassar W *et al*. [44]	Clinical, non-randomized trial	Stage 3–4 CKD patients	Intervention: cell-free cord-blood mesenchymal stem cells derived extracellular vesicles. Outcome: TGF-β1, IL-10, TNF-α plasma levels	Cell-free cord-blood mesenchymal stem cells derived extracellular vesicles (CF-CB-MSCs-EVs) are safe and can ameliorate the inflammatory immune reaction and improve the overall kidney function in stage 3–4 CKD patients
Ata A *et al*. [73]	Clinical, cross-sectional	Adults with ESKD related to hypertension and diabetes	Circulating miR-126-3p, miR-21-5p, miR-192-5p (EV-linked/plasma)	ESKD patients show decreased miR-126-3p and increased miR-21-5p and miR-192-5p, a profile compatible with pro-atherogenic and pro-inflammatory cardiovascular risk
de Boer HC *et al*. [28]	Clinical, intervention (aspirin)	Adults with type 2 diabetes and vascular disease	Platelet-related EVs and miR-126	In conditions with high platelet activation, aspirin lowers circulating miR-126, indicating that antiplatelet therapy modulates EV-miRNA profiles relevant for vascular risk (important when interpreting EV biomarkers in CKD with diabetes)
Behrens F *et al*. [61]	Clinical, observational (paediatric)	Children with CKD at different stages vs healthy controls	Endothelial-, platelet-, leukocyte- and erythrocyte-derived EVs; EV cargo	Paediatric CKD alters EV release and composition, impairs angiogenesis and promotes vascular pathology, suggesting EV involvement in very early cardiovascular damage
Liu P *et al*. [74]	Clinical, randomized cross-over	ESKD patients undergoing different dialysis modalities (HD vs HDF)	Platelet- and endothelial-derived EVs	EVs increase during HD and HDF; patterns differ between modalities, indicating that intradialytic cardiovascular stress and dialysis strategy both shape EV responses and potentially acute vascular risk
Mause SF *et al*. [63]	Clinical, interventional	Heart failure patients (substantial CKD burden) receiving intravenous iron	EMVs	Intravenous iron causes transient endothelial dysfunction and EMV release; CKD patients have impaired vascular repair, highlighting EV-mediated endothelial stress as a contributor to cardiovascular risk
Burrello J *et al*. [56]	Clinical, observational with machine learning	Adults across a cardiovascular-risk spectrum (including CKD)	Multiparametric EV surface antigen profiling (multiple cellular origins)	An EV surface-antigen ‘signature’ enables cardiovascular risk stratification and correlates with organ damage, suggesting that multiplex EV profiling can refine risk assessment in CKD beyond single markers
Zietzer A *et al*. [50]	Clinical, observational	Adults with coronary artery disease and CKD vs controls	Circulating EV-associated miRNAs (e.g. miR-223 and others)	CKD alters EV-miRNA-mediated vascular communication in CAD, promoting endothelial dysfunction and providing a mechanistic link between CKD and excess cardiovascular risk
Scullion KM *et al.* [76]	Clinical, longitudinal	ANCA-associated vasculitis, AKI/CKD and ESKD	Circulating Argonaute-bound miR-126 (largely EV-linked)	miR-126 is reduced in active vasculitis and ESKD and increases after treatment but remains low, indicating that miR-126-related EV signalling tracks vascular inflammation and vascular health across CKD stages
Carmona A *et al.* [48]	Clinical, observational	CKD patients	miRNAs expression profile	CKD patients present a specific circulating miRNAs expression profile associated with the microinflammatory state. Furthermore, microvesicles generated by monocytes treated with uraemic toxins induce early senescence and osteogenic markers (BMP2 and miRNA-223-3p) in VSMC
Jalal D *et al*. [51]	Clinical, observational	CKD patients vs non-CKD controls	Plasma proteins and EV-associated inflammatory/angiogenic markers	CKD patients show increased angiogenic proteins in plasma and inflammatory proteins within EVs, consistent with an EV-centred inflammatory/angiogenic milieu that favours cardiovascular disease
Nandula SR *et al*. [77]	Clinical, interventional (SGLT2 inhibitor)	Type 2 diabetes with CKD	CD34⁺ endothelial progenitor cell (EPC) function; podocyte exosome markers	Canagliflozin improves EPC function, reduces podocyte exosome markers and improves cardiovascular risk factors, indicating that SGLT2 inhibition beneficially modulates kidney- and vascular-derived EV signatures
Uil M *et al*. [54]	Clinical, cross-sectional	Type 2 diabetes with normo-, micro- or macro-albuminuria	Circulating EVs by cellular origin; miRNA cargo (e.g. miR-99a-5p)	Progressive diabetic nephropathy shows stage-dependent EV and miRNA profiles, linking CKD stage and albuminuria to altered vascular EV signalling
Fonseca F *et al*. [52]	Clinical, cross-sectional	Advanced CKD vs controls	Monocyte-derived microparticles	Advanced CKD is associated with higher monocyte microparticle concentrations, supporting a role for myeloid EVs in the inflammatory vascular phenotype and elevated cardiovascular risk of CKD
Cavallari C *et al*. [78]	Clinical, randomized clinical trial comparing HDF and bicarbonate HD	ESKD randomly switched to HDF or maintained on bicarbonate HD	miR-223 expression in plasma EVs	The switch from bicarbonate HD to mixed online HDF reduced systemic inflammation and miR-223 expression in plasma EV, improving *in vitro* angiogenesis and reducing vascular calcification
Barutta F *et al*. [79]	Clinical, cross-sectional	Type 1 diabetic patients with and without incipient diabetic nephropathy	miR-130, miR-145, miR-155 and miR-424 in urinary exosomes in microalbuminuric vs non-microalbuminuric patients	miR-130a and miR-145 were enriched, while miR-155 and miR-424 reduced in urinary exosomes from patients with microalbuminuria

EGFR, endothelial growth factor receptor; ALP, alkaline phosphatase; HD, haemodialysis; HDF, haemodiafiltration; EMV, endothelial microvesicles; AKI, acute kidney injury; SGLT2, sodium-glucose cotransporter 2.

The accumulation of uraemic toxins, chronic inflammation, oxidative stress, and profound disturbances in mineral metabolism and vascular calcification characterize CKD. Uraemic toxins such as indoxyl sulphate and p-cresyl sulphate have been shown to directly induce endothelial cell activation and apoptosis, leading to increased shedding of pro-inflammatory and pro-coagulant EVs [45, 46]. Oxidative stress-related EVs exert both beneficial and harmful effects, as they can carry antioxidants, reactive oxygen species (ROS)-generating enzymes and oxidized molecules [47]. Inflammatory cytokines [48] and oxidative stress [47] further amplify EV release, creating a feed-forward loop that perpetuates vascular injury.

In CKD, circulating EVs are not only increased in number but also altered in their cellular origin and molecular cargo, reflecting and propagating the unique metabolic, inflammatory and pro-calcific milieu of renal dysfunction [10, 49]. MicroRNA-mediated intercellular communication in the vasculature is altered in CKD, which promotes endothelial dysfunction [50]. CKD patients show increased plasma levels of angiogenic proteins and inflammatory proteins in EVs, indicating activated vascular injury pathways [51]. Advanced CKD is associated with higher circulating monocyte microparticle levels [52]. Plasma-derived mediate glomerular endothelial cell injury in diabetic nephropathy in rats [53] and in patients with overt diabetic nephropathy platelet-derived EVs in plasma are upregulated [54]. On the other hand, in patients with CKD, circulating levels of miR-126 are reduced, and this reduction is associated with endothelial dysfunction and increased cardiovascular risk [55, 56]. In theory, EVs surface antigen profiling might have potential for CV risk stratification because specific EVs markers correlate with CV risk factors and organ damage [56]. However, appropriate prognostic studies are still lacking.

Disturbances in mineral metabolism, particularly hyperphosphataemia and secondary hyperparathyroidism, are common in CKD and drive vascular calcification [5]—a process in which EVs play a pivotal role [57]. VSMC exposed to high phosphate levels release EVs that serve as nucleation sites for hydroxyapatite crystal formation. These calcifying EVs are enriched in annexins, fetuin-A and specific microRNAs that promote mineral deposition and inhibit natural calcification inhibitors. The result is increased arterial stiffness, reduced vascular compliance and heightened risk of left ventricular hypertrophy and heart failure. Interestingly, activation of IκB kinase 2 (IKK2)/nuclear factor (NF)-κB in VSMCs protects against CKD-induced vascular calcification by reducing apoptotic, calcifying EVs [58]. Furthermore, epidermal growth factor receptor inhibition prevents the biogenesis of vascular calcifying EVs [59].

CKD is a state of chronic, low-grade inflammation [3]. EVs derived from activated leukocytes and platelets in CKD patients are loaded with inflammatory cytokines [e.g. interleukin (IL)-1β, TNF-α] and pro-inflammatory microRNAs [49]. These EVs can induce endothelial activation, promote monocyte adhesion and transmigration, and propagate immune cell activation within the vascular wall. This persistent inflammatory signalling not only accelerates atherosclerosis but also contributes to plaque instability and the risk of acute coronary events.

CKD alters miRs-mediated intercellular communication via EVs in patients with coronary artery disease (CAD) [50]. EVs in patients treated with haemodiafiltration had lower EV-miR-223 levels than EVs in patients on bicarbonate-based haemodialysis [57], a mechanism that might improve angiogenesis, decrease endothelial cell apoptosis and reduce VSMC calcification. Increased levels of EC-derived EVs were also observed in diabetic kidney disease patients (as well as increased EV levels derived from platelets, leukocytes and erythrocytes) [60], and in CKD patients with CAD [50, 61].

Importantly, EVs in CKD exert direct effects on the myocardium. Dysregulated EVs cargo can be taken up by cardiac fibroblasts and cardiomyocytes, promoting maladaptive gene expression, hypertrophy and fibrosis [62]. Experimental studies have shown that EVs from CKD patients induce profibrotic signalling pathways and impair mitochondrial function in cardiac cells, contributing to the development of uraemic cardiomyopathy—a distinct form of heart disease characterized by left ventricular hypertrophy, diastolic dysfunction and interstitial fibrosis. Remarkably, intravenous iron supplementation in heart failure patients induces temporary endothelial dysfunction, accompanied by the release of endothelial microvesicles [63]. Inhibition of sodium-glucose transporter-2 by empagliflozin enhanced exosome secretion from bone marrow-derived MSCs. Both empagliflozin and untreated exosomes attenuated myocardial ischaemia–reperfusion injury. Notably, the association of empagliflozin and untreated exosomes had superior therapeutic efficacy compared to untreated exosomes, suggesting a potential strategy for optimizing MSC-derived exosome therapy in ischaemic heart disease [64]. Sodium-glucose transporter-2 inhibition in patients with CKD and diabetes reduces podocyte exosome markers [65] and has well-documented beneficial effects on cardiovascular risk [66].

CKD patients are at increased risk of thrombotic events, including MI and stroke [2]. EVs from platelets and endothelial cells in CKD are enriched in tissue factor and phosphatidylserine, providing a catalytic surface for activation of the coagulation cascade [67]. This prothrombotic EVs profile is further exacerbated by impaired fibrinolysis and heightened platelet reactivity, creating a milieu that favours both arterial and venous thrombosis.

**Figure 2:**
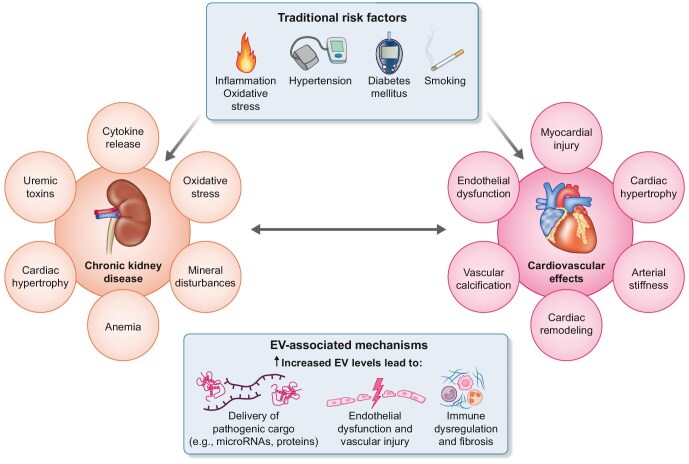
The figure shows how EVs bridge CKD and CVD. CKD-dependent risk factors (left panel) and traditional risk factors (upper panel) contribute to causing cardiovascular alterations, from cardiac remodelling to vascular calcification (right panel). The mechanisms by which EVs cause kidney and cardiovascular damage include delivery of pathogenic EVs cargo (e.g. microRNAs and proteins), endothelial dysfunction, altered immune regulation and fibrosis.

The unique EVs profile in CKD and end-stage kidney disease (ESKD) patients not only reflects but actively drives the high cardiovascular risk in this population. EVs are emerging as promising biomarkers for early detection and risk stratification of CVD in these populations, with several studies demonstrating correlations between EVs subtypes or cargo and cardiovascular outcomes [63, 68]. Furthermore, targeting EVs biogenesis, release or uptake represents a novel therapeutic strategy to interrupt maladaptive signalling pathways. The interplay of CKD-specific factors, EVs biology and cardiovascular pathology is illustrated in Fig. 2.

## A ROADMAP TO THE THERAPEUTIC POTENTIAL OF EVs IN CKD-ASSOCIATED CVD

The clinical and translational potential of EVs in CKD-associated CVD is summarized in Table 2.

**Table 2:** ‘Roadmap’ elements in preclinical or clinical studies discussed in the review and separate potential therapeutic applications from upstream mechanistic evidence.

**Level of evidence/stage**	**EV-based strategy**	**Main supporting studies [Ref.]**	**Target process in CKD-associated CVD**	**Current status and key gaps**
Preclinical—mechanistic vascular	Modulating calcifying EVs from vascular smooth muscle cells via IKK2/NF-κB activation or EGFR inhibition	Miyazaki-Anzai *et al*. [58]; Bakhshian Nik *et al*. [57]	VSMC-derived calcifying EVs that nucleate hydroxyapatite and drive medial calcification and arterial stiffness in CKD	Robust animal and *in vitro* data show that signalling pathways (IKK2/NF-κB, EGFR) regulate calcifying EV biogenesis, but there are no human interventional studies targeting these axes yet
Preclinical—mechanistic endothelial	Limiting uraemic toxin–induced endothelial dysfunction by targeting EC-derived EVs	Figuer *et al*. [46]; studies on indoxyl sulfate and oxidative stress-driven EV changes [45–47]	Indoxyl sulfate– and ROS-driven release of pro-inflammatory, pro-coagulant EC-EVs that amplify endothelial injury	Mechanistic *in vitro* work links specific toxins to dysfunctional EC-EVs; translation requires identifying druggable steps in EV release or cargo loading and testing them *in vivo*
Preclinical—mechanistic inflammatory/thrombotic	Modifying leukocyte- and platelet-derived EVs that promote inflammation, coagulation and atherosclerosis	Buffolo *et al*. [20]; Jansen *et al*. [22]; Berezin *et al*. [49]; Jalal *et al*. [51]; Fonseca *et al*. [52]; Bakhshian Nik *et al*. [57]	EV-mediated recruitment and activation of leukocytes, monocytes and platelets; tissue factor–rich EVs favouring thrombosis in CKD	Strong pathophysiological rationale and association data, but no specific EV-directed anti-thrombotic or anti-inflammatory therapies have been tested clinically in CKD
Preclinical—translational, cardiac repair	MSC-derived EVs and engineered EVs to protect and repair the myocardium	Zhao *et al*. [38]; Zhu *et al*. [39]; Liu *et al*. [40]; Nassar *et al*. [44]	Attenuation of myocardial ischaemia–reperfusion injury, reduction of apoptosis, improved post-infarction remodelling via miR-182, miR-125b, miR-93-5p and related cargo	Multiple animal models show cardioprotection, including in kidney-relevant contexts; translation to CKD patients with CVD awaits standardized production, dosing and safety data
Preclinical—translational, vascular repair	Endothelial EVs enriched in miR-126/miR-222 to promote endothelial repair and angiogenesis	Jansen *et al*. [34]; Li *et al*. [37]	Re-endothelialization after injury, improved endothelial function and angiogenesis via EV-mediated transfer of protective miRNAs	Proof-of-concept *in vitro* and *in vivo*; needs adaptation to CKD, where baseline endothelial dysfunction and uraemic toxins may alter EV efficacy and biodistribution
Clinical—modulation by dialysis and CKD care	Using dialysis modality and extracorporeal strategies to favour a less injurious EV profile (e.g. online HDF, optimized HD)	Liu P *et al*. [74]; Cavallari *et al*. [78]; Zietzer *et al*. [50]; Behrens *et al*. [61]	Inflammation-related endothelial dysfunction, vascular calcification and EV-miRNA alterations (e.g. miR-223) in ESKD	Human data show that dialysis modality and membrane choice change EV counts and miRNA cargo; whether modifying EVs in this way improves hard CV endpoints in CKD remains untested
Clinical—drug-mediated EV modulation	Leveraging or avoiding EV changes induced by existing drugs (aspirin, statins, SGLT2 inhibitors, IV iron)	de Boer *et al*. [28]; Kulshreshtha *et al*. [69]; Sun *et al*. [70]; Zietzer *et al*. [50]; Mause *et al*. [63]; Nandula *et al*. [77]; Hannouneh *et al*. [64]	Aspirin-induced shifts in platelet EV miR-126; statin- and PCSK9-related effects on EV content; SGLT2i-related changes in podocyte and vascular EVs; IV iron-induced EMV surges	These agents already used in CKD and CVD clearly modulate EVs; however, current trials were not designed to test whether EV changes mediate cardiovascular benefit or harm, and EVs are not yet used to guide therapy
Clinical—early EV-based interventions	Cell-free MSC therapy and first-in-human exosome trials as a template for future EV-based CKD–CVD interventions	De Stefano [42, Nassar *et al*. 44]; Kalluri *et al*. [72]	Renal function in stage 3–4 CKD; safety and feasibility of exosome therapy in other indications	MSC therapy in CKD and exosome trials in non-CKD populations demonstrate feasibility and short-term safety; specific EV-based interventions for CKD-associated CVD have not yet entered clinical testing
Conceptual—future CKD–CVD EV trials	Designing randomized trials of EV-based products or EV-guided strategies in CKD patients with high cardiovascular risk	Roadmap section [this review]; supported by preclinical cardiac and vascular EV studies [38–40, 55–59, 63]	Cardiovascular death, HF hospitalization, MI, surrogate vascular measures (arterial stiffness, calcification, endothelial function)	At present, EV-based therapy in CKD-associated CVD is a conceptual roadmap. Rigorous GMP-grade EV production, potency assays, dosing, and controlled phase 1–2 trials in carefully selected CKD–CVD populations are needed before large outcome trials can be justified

In this table we explicitly identify each ‘roadmap’ element in preclinical or clinical studies discussed in the review and separate potential therapeutic applications from upstream mechanistic evidence. Therapeutic applications (MSC EVs, endothelial EVs, clinical trials) are kept in rows where there is a genuine therapeutic angle, while purely mechanistic issues (uraemic toxin effects, calcifying VSMC EVs) are separately presented in mechanistic rows. The roadmap is still conceptual. EGFR, endothelial growth factor receptor; SGLT2, sodium-glucose cotransporter 2; IV, intravenous; HF, heart failure; GMP, Good Manufacturing Practices.

EVs are being explored as delivery vehicles for therapeutic agents due to their ability to cross biological membranes efficiently. Engineered EVs and sustained-release systems, such as hydrogel patches, have shown promise in preclinical models for enhancing cardiac repair and function after MI. However, translating these findings into clinical practice requires rigorous standardization of EV production, characterization and delivery methods, as well as robust evidence of safety and efficacy.

Immunogenicity is a central consideration for the therapeutic use of EVs, because it underpins both safety and pharmacokinetics. Native EVs, especially those derived from human cells, generally display high biocompatibility and relatively low immunogenicity compared with synthetic nanoparticles or viral vectors: they are non‑replicating, lack viral proteins, and present ‘self‑like’ membrane components that limit overt immune activation and allow more prolonged circulation and tissue distribution [69]. At the same time, EVs are not immunologically inert. Their surface proteins, lipids, glycosylation patterns and biomolecular corona, as well as their cellular origin, production methods, dose and route of administration, can all influence immune recognition, clearance and, in some contexts, unintended immunostimulation [69].

Established cardiovascular drugs such as aspirin [28, 43] and statins [49, 70] modulate EVs, thereby contributing to their therapeutic efficacy. These agents do not merely exert their effects through classical pathways—such as platelet inhibition or lipid lowering—but also influence the biogenesis, release and molecular cargo of EVs derived from various cardiovascular and immune cells. For instance, statins have been shown to alter the miRNA and protein composition of EVs released from endothelial and progenitor cells, enhancing their anti-inflammatory and reparative properties [72]. This modulation can attenuate endothelial dysfunction and vascular inflammation, both of which are central to atherogenesis and plaque instability. Moreover, statin-induced EVs may carry specific miRNAs that suppress pro-inflammatory gene expression in recipient cells, thereby exerting systemic anti-atherogenic effects beyond cholesterol lowering [70, 72].

Human trials of EV-based therapies for cardiovascular complications in CKD are still lacking. Challenges related to reproducibility, scalability and regulatory standards should be addressed before EVs can be established as reliable diagnostic biomarkers or therapeutic agents in clinical settings. This would require the design of *in vitro* assays to assess therapeutic potency.

To translate the encouraging preclinical findings into clinical benefit, a carefully structured, scientifically sound approach is required for designing and conducting human studies. The initial step in human research would focus on safety and feasibility. The safety and feasibility of exosome therapy have already been established in a phase 1 clinical trial authorized by the Food and Drug Administration [73]. Early-phase clinical trials would enroll CKD patients with stable chronic heart failure or those recovering from a recent MI, populations that are at risk but stable enough to participate in first-in-human studies. These trials would typically use an open-label, dose-escalation design, carefully monitoring participants for adverse events, immune reactions and laboratory abnormalities. The primary aim at this stage is to establish that EVs administration is safe and well-tolerated, while also gathering preliminary data on potential efficacy through biomarkers and imaging studies.

If safety is confirmed, subsequent studies would expand to assess preliminary efficacy in a larger, more diverse patient population. Here, randomized, double-blind, placebo-controlled trials would be appropriate, with patients assigned to receive either EVs therapy or placebo. The intervention could be delivered intravenously or directly into the coronary arteries, depending on the clinical context and the disease stage. Outcomes would focus on changes in cardiac function, such as improvements in left ventricular ejection fraction, reductions in biomarkers like NT-proBNP, and enhancements in patient quality of life and exercise capacity. These studies would also track major adverse cardiovascular events to detect any signals of clinical benefit or harm.

As evidence accumulates, larger, multicentre trials would be needed to confirm efficacy and safety over more extended periods and in broader populations. These studies would use rigorous endpoints, such as cardiovascular death, heart failure hospitalizations, and sustained improvements in functional and structural cardiac measures. Throughout all phases, the source and characterization of EVs must adhere to Good Manufacturing Practice standards, with strict quality control to ensure consistency and safety. The route and timing of administration should be optimized based on the specific clinical scenario, and patient selection criteria should be carefully defined to minimize confounding factors.

In parallel, mechanistic substudies using biomarkers and advanced imaging would help elucidate how EVs exert their effects and identify which patients are most likely to benefit. Safety monitoring must remain vigilant, with particular attention to immune responses and potential arrhythmic risks. Regulatory and ethical oversight is paramount, requiring early and ongoing engagement with health authorities and ethics committees to ensure that all studies meet the highest standards of patient safety and scientific integrity.

The ability to produce large quantities of high-quality EVs, maintain batch-to-batch consistency and monitor for late adverse events will be critical for successful translation. Over time, there may also be opportunities to personalize EVs therapy based on individual patient characteristics or disease phenotypes.

## CONCLUSIONS AND PERSPECTIVES

EVs have emerged as important mediators in the complex interplay between CKD and cardiovascular complications such as coronary heart disease and cardiomyopathy. Far from being passive byproducts, EVs orchestrate key pathogenic processes—including endothelial dysfunction, vascular calcification, inflammation, cardiac remodelling and thrombosis—thereby providing a mechanistic link between renal impairment and cardiovascular disease. Their unique capacity to function both as biomarkers and as active effectors of disease progression underscores their potential as targets for innovative diagnostic and therapeutic strategies. As our understanding of EVs biology deepens, harnessing their properties may pave the way for new approaches that could impact the management of CKD-associated cardiovascular disease, ultimately improving patient outcomes.

## Data Availability

There are no new data associated with this article.
